# A Spider-Derived Kunitz-Type Serine Protease Inhibitor That Acts as a Plasmin Inhibitor and an Elastase Inhibitor

**DOI:** 10.1371/journal.pone.0053343

**Published:** 2013-01-04

**Authors:** Hu Wan, Kwang Sik Lee, Bo Yeon Kim, Feng Ming Zou, Hyung Joo Yoon, Yeon Ho Je, Jianhong Li, Byung Rae Jin

**Affiliations:** 1 College of Natural Resources and Life Science, Dong-A University, Busan, Republic of Korea; 2 Department of Agricultural Biology, National Academy of Agricultural Science, Suwon, Republic of Korea; 3 Department of Agricultural Biotechnology, Seoul National University, Seoul, Republic of Korea; 4 Department of Plant Protection, Huazhong Agricultural University, Wuhan, PR China; Leiden University Medical Center, The Netherlands

## Abstract

Kunitz-type serine protease inhibitors are involved in various physiological processes, such as ion channel blocking, blood coagulation, fibrinolysis, and inflammation. While spider-derived Kunitz-type proteins show activity in trypsin or chymotrypsin inhibition and K^+^ channel blocking, no additional role for these proteins has been elucidated. In this study, we identified the first spider (*Araneus ventricosus*) Kunitz-type serine protease inhibitor (AvKTI) that acts as a plasmin inhibitor and an elastase inhibitor. AvKTI possesses a Kunitz domain consisting of a 57-amino-acid mature peptide that displays features consistent with Kunitz-type inhibitors, including six conserved cysteine residues and a P1 lysine residue. Recombinant AvKTI, expressed in baculovirus-infected insect cells, showed a dual inhibitory activity against trypsin (K_i_ 7.34 nM) and chymotrypsin (K_i_ 37.75 nM), defining a role for AvKTI as a spider-derived Kunitz-type serine protease inhibitor. Additionally, AvKTI showed no detectable inhibitory effects on factor Xa, thrombin, or tissue plasminogen activator; however, AvKTI inhibited plasmin (K_i_ 4.89 nM) and neutrophil elastase (K_i_ 169.07 nM), indicating that it acts as an antifibrinolytic factor and an antielastolytic factor. These findings constitute molecular evidence that AvKTI acts as a plasmin inhibitor and an elastase inhibitor and also provide a novel view of the functions of a spider-derived Kunitz-type serine protease inhibitor.

## Introduction

Kunitz-type serine protease inhibitors are ubiquitous, exist in multiple forms, and are found in numerous tissues of many organisms, including animals, plants, and microbes. These protease inhibitors consist of approximately 60 amino acid residues that display features such as three disulfide bridges, which contribute to the stable nature of the folded mature peptide, and a P1 site, which corresponds to the specificity of their cognate enzymes [1]–[3]. Functionally, Kunitz-type serine protease inhibitors show inhibitory activity against trypsin [4]–[8], chymotrypsin [9]–[11], or both [12]. Many Kunitz-type serine protease inhibitors have been isolated from blood-sucking and venomous animals, including snakes [2], [4], [7], [9], [10], [12], bees [7], wasps [4], cattle ticks [8], [13], frogs [11], sea anemones [6], scorpions [14], black flies [15], and spiders [3], [16]. Kunitz-type serine protease inhibitors are involved in various physiological processes, such as ion channel blocking, blood coagulation, fibrinolysis, and inflammation [3], [7], [17]–[21]. Thus, these Kunitz-type serine protease inhibitors appear to have a strong potential for pharmaceutical development [7], [14], [17]–[21].

A recent publication has categorized Kunitz-type proteins into five classes: body trypsin inhibitors, chymotrypsin inhibitors in venom, trypsin inhibitors in venom, double-functional toxins, and potassium channel blockers [3]. Among spider species, Kunitz-type serine protease inhibitors from tarantulas are the best studied [3], [16]. Although spider Kunitz-type serine protease inhibitors have been isolated and characterized, the roles of these inhibitors, with the exceptions of chymotrypsin or trypsin inhibition and potassium channel blocking [3], [16], remain relatively unexplored. In snake venom, Kunitz-type serine protease inhibitors demonstrate antifibrinolytic activity [17]–[19], [21]. Tick-derived Kunitz-type serine protease inhibitors function as antihemostatic factors [20]. Additionally, our previous study provided evidence for an antifibrinolytic role of a bumblebee venom Kunitz-type serine protease inhibitor, which acts as a plasmin inhibitor [7]. Two Kunitz family proteins from the salivary glands of black fly inhibit enzymes that regulate clotting and inflammatory responses [15]. Until now, the antifibrinolytic activity and/or antielastolytic activity of spider-derived Kunitz-type serine protease inhibitors has not been determined.

The objective of this study is to further elucidate the functions of spider-derived Kunitz-type serine protease inhibitors. We report the first spider-derived Kunitz-type serine protease inhibitor that acts as an antifibrinolytic factor and an antielastolytic factor. Our results describe the molecular characterization of a spider (*Araneus ventricosus*) Kunitz-type serine protease inhibitor (AvKTI) that exhibits inhibitory activity against trypsin, chymotrypsin, plasmin, and neutrophil elastase.

## Results

### AvKTI is a Spider Kunitz-type Serine Protease Inhibitor

**Figure 1:**
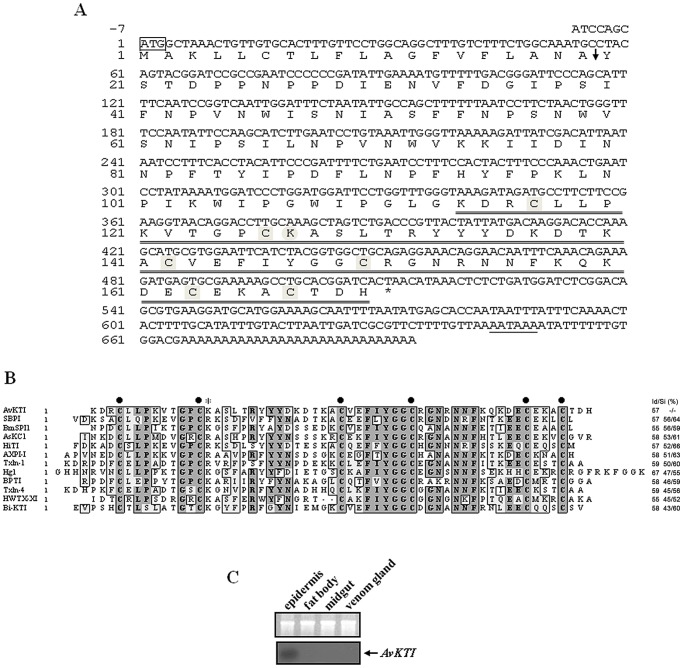
AvKTI is a Kunitz-type serine protease inhibitor. (A) The nucleotide and deduced amino acid sequences of *AvKTI* cDNA (GenBank accession no. JX844659). The start codon (ATG) is boxed, and the termination codon is indicated with an asterisk. The putative polyadenylation signal is underlined. The predicted signal sequence, a pro-peptide, and the mature peptide are indicated. The characteristic cysteine residues are indicated by squares. The P1 position is marked with a circle. (B) The alignment of the amino acid sequences for mature AvKTI with other known Kunitz-type serine protease inhibitors. The characteristic cysteine residues are shown in bold. The P1 position is marked with an asterisk. The sources of the aligned sequences were *A. ventricosus* (this study, GenBank accession no. JX844659), *Sarcophaga bullata* SBPI (P26228), *Bombyx mori* BmSPI1 (NP_001037044), *Anemonia sulcata* AsKC1 (Q9TWG0), *Haematobia irritans irritans* HiTI (AAL87009), *Anthopleura aff. xanthogrammica* AXPI-I (P81547), *Pseudonaja textilis textilis* Txln-1 (Q90WA1), *Hadrurus gertschi* Hg1 (P0C8W3), *Bos taurus* BPTI (P00974), *Pseudonaja textilis textilis* Txln-4 (Q90W98), *Haplopelma schmidti* HWTX-XI (P68425), and *Bombus ignitus* Bi-KTI (AEM68408). The AvKTI sequence was used as a reference for the identity/similarity (Id/Si) values. (C) Expression of *AvKTI* in *A. ventricosus*. Total RNA was isolated from the epidermis, fat body, silk gland, and venom gland of *A. ventricosus*. RNA was separated by 1.2% formaldehyde agarose gel electrophoresis, transferred onto a nylon membrane, and hybridized with radiolabeled *AvKTI* cDNA (lower panel). *AvKTI* transcripts are indicated with an arrow. The ethidium bromide-stained RNA gel shows uniform loading (upper panel).

To characterize the spider-derived Kunitz-type serine protease inhibitor, we identified an EST for a gene encoding a Kunitz-type serine protease inhibitor (AvKTI) in an *A. ventricosus* cDNA library. An *AvKTI* cDNA that included the full-length Kunitz-type serine protease inhibitor gene was identified by searching the *A. ventricosus* ESTs (GenBank accession number JX844659). Database searches using the predicted AvKTI sequence indicated that the AvKTI sequence contained a Kunitz domain, which is a feature of Kunitz family protease inhibitors (Figures 1A and B). We assigned the pro-peptide region based on the signal peptide identified by the SignalP program and the mature peptide predicted by alignment with other Kunitz-type inhibitors. AvKTI consisted of 170 amino acids, which included a 19-amino acid signal peptide, a 94-amino acid pro-peptide, and a 57-amino acid mature peptide (Figure 1A). Analysis of the peptide sequence of mature AvKTI showed similarity to members of other Kunitz-type serine protease inhibitor families that display distinctive features, including six conserved cysteine residues and a P1 site (Figure 1B). These features suggest that AvKTI is structurally and functionally similar to other Kunitz-type serine protease inhibitors.

We examined the expression pattern of *AvKTI* in *A. ventricosus* to confirm that it is an *A. ventricosus*-derived Kunitz-type serine protease inhibitor. Northern blot analysis showed that *AvKTI* was expressed only in the epidermis (Figure 1C).

### AvKTI Inhibits Trypsin and Chymotrypsin

**Figure 2:**
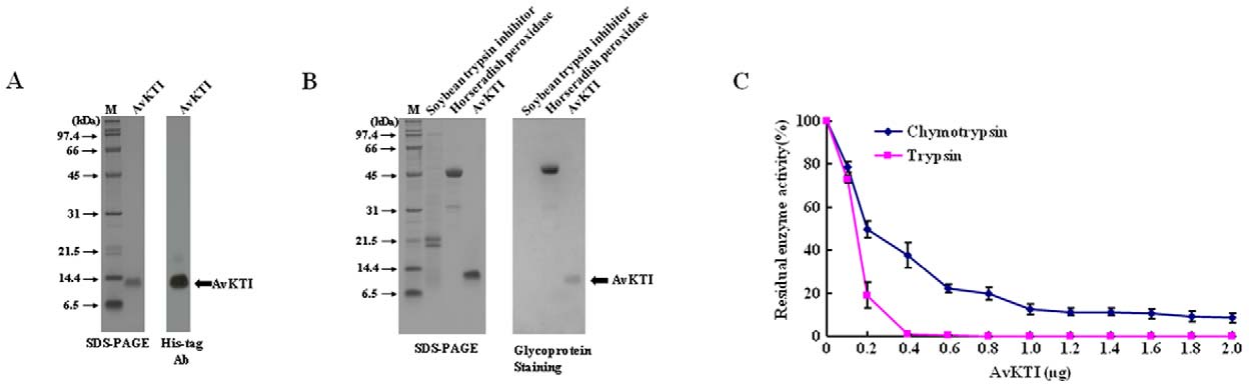
AvKTI is *O*-glycosylated and inhibits trypsin and chymotrypsin. (A) SDS-PAGE (left) and western blot analysis (right) of purified recombinant AvKTI expressed in baculovirus-infected Sf9 insect cells. Recombinant AvKTI was identified using a His-tag antibody. (B) Glycoprotein staining of AvKTI. Purified AvKTI and control protein samples were subjected to 12% SDS-PAGE (left) and then analyzed by glycoprotein staining (right). Horseradish peroxidase (5 µg), a glycosylated protein, was used as a positive control. Soybean trypsin inhibitor (5 µg), a non-glycosylated protein, was used as a negative control. (C) Enzyme inhibition by AvKTI. Trypsin or chymotrypsin was incubated with increasing amounts of AvKTI, and the residual enzyme activity was then determined (*n* = 3).

To further characterize AvKTI, we expressed the mature peptide of AvKTI in baculovirus-infected insect cells. The purified recombinant AvKTI, which contained an additional 6 His residues, was present as a 13-kDa protein (Figure 2A). However, the molecular mass of AvKTI expressed in insect cells was much larger than the predicted molecular mass of AvKTI (7.2 kDa). Several putative *O*-glycosylation sites, but no *N*-glycosylation sites, were found in the sequence of the mature peptide of AvKTI. To determine whether AvKTI was indeed glycosylated, glycoprotein staining of the recombinant AvKTI was performed. The result shows that the difference between the predicted molecular mass of 7.2 kDa for the mature peptide of AvKTI and the molecular mass of 13 kDa obtained by SDS-PAGE was due to the presence of carbohydrate moieties (Figure 2B).

Using recombinant AvKTI, we investigated the inhibitory effects of the peptide. AvKTI exhibited inhibitory activity against trypsin and chymotrypsin (Figure 2C), indicating that AvKTI has a dual inhibitory function against trypsin and chymotrypsin, although the inhibitory ability against chymotrypsin (IC_50_: 109.25 nM) was 2.5-fold weaker than that against trypsin (IC_50_: 43.39 nM) (Table 1). In this experiment, the inhibitory constants (K_i_) of AvKTI against trypsin and chymotrypsin were 7.34 nM and 37.75 nM, respectively (Table 1).

**Table 1:** The inhibitory activities of AvKTI against serine proteases.

Enzyme	Concentration (nM)^a^	IC_50_ (nM)	Ratio^b^	K_i_ (nM)
Trypsin	100	43.39	0.43	7.34
α-Chymotrypsin	100	109.25	1.09	37.75
Plasmin	100	10.07	0.10	4.89
Neutrophil elastase	100	446.93	4.47	169.07

a The concentration of enzyme used in this experiment.

b The molar ratio of IC_50_ to the enzyme concentration.

### AvKTI Acts as a Plasmin Inhibitor and an Elastase Inhibitor

**Figure 3:**
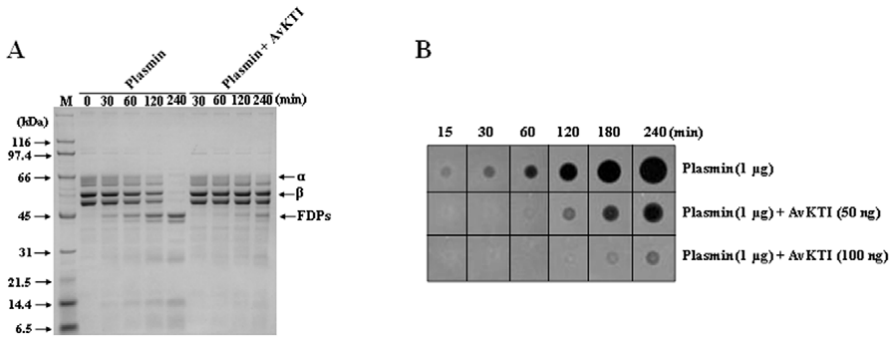
AvKTI exhibits antifibrinolytic activity. (A) AvKTI-mediated plasmin inhibition assay. The number indicates the time (in minutes) that fibrin was incubated with plasmin or both plasmin and AvKTI. The FDPs are shown. (B) The antifibrinolytic activity of AvKTI. Plasmin was dropped onto fibrin plates along with different amounts of AvKTI, and the plates were then incubated at 37°C for various periods of time.

Given that AvKTI is a Kunitz-type inhibitor, we investigated whether AvKTI functions similarly to other Kunitz-type serine protease inhibitors that exhibit antifibrinolytic activity [7], [15], [17]–[19] and/or antielastolytic activity [8], [15]. Based on an analysis of the degradation of human fibrin by plasmin over time, we observed that AvKTI significantly inhibited the transformation of fibrin to fibrin degradation products (FDPs) (Figure 3A). Subsequently, we assayed the antifibrinolytic activity of AvKTI on a fibrin plate. This experiment showed that the addition of AvKTI inhibited the plasmin-mediated formation of a clear area in a dose- and time-dependent manner (Figure 3B). Our results show that AvKTI inhibits the plasmin-mediated degradation of fibrin to FDPs, which is consistent with an antifibrinolytic activity for AvKTI.

**Figure 4:**
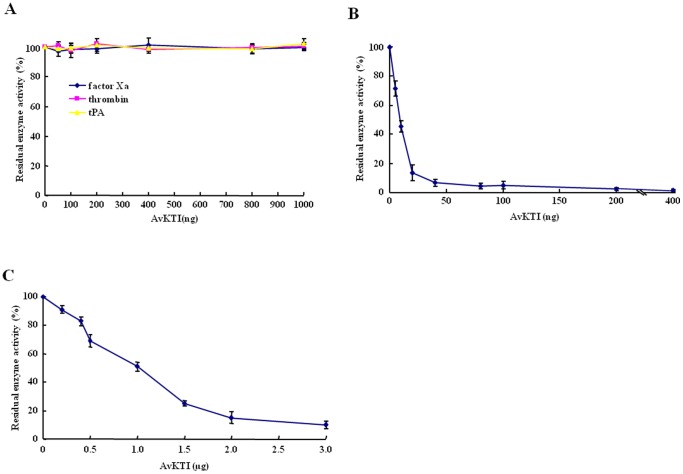
AvKTI inhibits plasmin and elastase, but not factor Xa, thrombin, or tPA. (A) Inhibitory activity of AvKTI against several enzymes associated with the hemostatic system. Factor Xa, thrombin, or tPA was incubated with increasing amounts of AvKTI, and the residual enzyme activity was determined (*n* = 3). (B, C) The inhibitory activities of AvKTI against plasmin (B) and neutrophil elastase (C). Plasmin or neutrophil elastase was incubated with increasing amounts of AvKTI, and the residual enzyme activity was determined (*n* = 3).

We then assessed whether AvKTI inhibits several other enzymes associated with the hemostatic system. AvKTI had no detectable inhibitory effect on factor Xa, thrombin, or tPA activity (Figure 4A). In contrast, AvKTI strongly inhibited plasmin (Figure 4B), indicating that AvKTI is a plasmin inhibitor. We also determined the inhibitory activity of AvKTI against neutrophil elastase and found that AvKTI inhibited neutrophil elastase (Figure 4C), indicating that AvKTI is an elastase inhibitor; however, the inhibitory ability of AvKTI against neutrophil elastase (IC_50_: 446.93 nM) was 44.4-fold weaker than that against plasmin (IC_50_: 10.07 nM). Based on the ratio of inhibitor to enzyme, AvKTI most strongly inhibited plasmin among the four enzymes used in this study (Table 1). The K_i_ of AvKTI against plasmin and neutrophil elastase was 4.89 nM and 169.07 nM, respectively (Table 1). These results show that AvKTI has antifibrinolytic and antielastolytic functions, thereby defining roles for AvKTI as a plasmin inhibitor and an elastase inhibitor.

**Figure 5:**
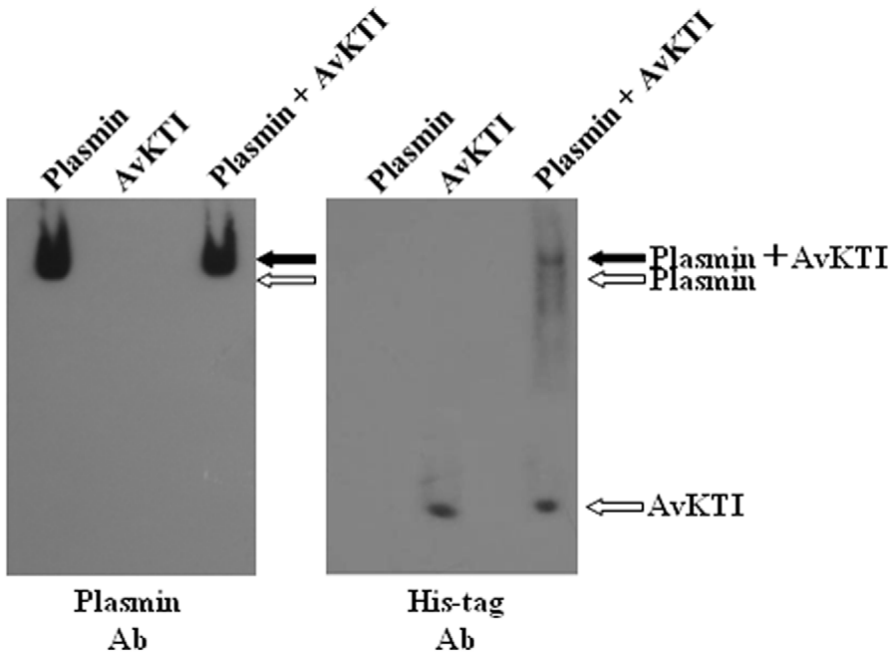
AvKTI forms complexes with plasmin. Western blot analysis of the formation of plasmin-AvKTI complex via native gel electrophoresis was performed. Three micrograms of plasmin were incubated with 1 µg of AvKTI, and the samples (plasmin, AvKTI, or plasmin-AvKTI mixture) were resolved on a 10% polyacrylamide gel. After electrophoresis, the protein samples were incubated with antiserum against plasmin or His-tag. The plasmin, AvKTI, and plasmin-AvKTI complex are indicated with arrows.

To study the mechanism by which AvKTI strongly inhibits plasmin, we assessed the formation of plasmin-AvKTI complexes using native gel electrophoresis followed by Western blotting. The electrophoretic mobility shift assay showed that AvKTI bound to plasmin, indicating the formation of plasmin-AvKTI complexes (Figure 5).

## Discussion

In addition to the inhibitory functions of serine proteases, such as against trypsin and/or chymotrypsin, some Kunitz family protease inhibitors are involved in the processes of coagulation, fibrinolysis, and inflammation [7], [8], [15], [17]–[19]. Therefore, many Kunitz-type serine protease inhibitors have been identified and characterized from various organisms. In tarantula spider species, a superfamily of Kunitz-type proteins has been discovered [3], [16]. Nonetheless, new functions of spider-derived Kunitz-type proteins have not been determined, with the exception of the trypsin or chymotrypsin inhibition and K^+^ channel blocking [3], [16].

In this study, we identified the first spider-derived Kunitz-type serine protease inhibitor that acts as a plasmin inhibitor and an elastase inhibitor. Based on its possession of the features of Kunitz-type serine protease inhibitors, including six cysteine residues and a P1 site [2], [3], we hypothesized that AvKTI is similar to Kunitz-type serine protease inhibitors. We found that AvKTI contains a potential signal peptide, the Kunitz domain of a mature peptide, and an intervening pro-peptide, as has been shown for several Kunitz-type proteins [3], [16]. However, the reason for the presence of an intervening pro-peptide that is 94-amino acids long in AvKTI remains unclear, but it is possible that AvKTI forms a precursor structure [16]. AvKTI is expressed only in the epidermis, suggesting that, based on the category of Kunitz-type proteins [3], AvKTI is a Kunitz-type serine protease inhibitor derived from the spider body, but not from venom. Additionally, AvKTI shares 56% protein sequence identity with other Kunitz-type protease inhibitors, such as *Sarcophaga bullata* SBP1, which is isolated from the larval hemolymph [22], and *Bombyx mori* BmSPI1, which is expressed in middle silk glands [23]. Future functional studies will be needed to characterize the physiological target and role of AvKTI in *A. ventricosus*.

Kunitz-type serine protease inhibitors show inhibitory activity against trypsin [4]–[7], chymotrypsin [9]–[11], or both [12]. We therefore expressed the mature peptide for AvKTI in baculovirus-infected insect cells and tested whether AvKTI inhibits trypsin and/or chymotrypsin. The AvKTI expressed in insect cells was *O*-glycosylated, as has been shown for the Kunitz-type serine protease inhibitor of black fly [15]. Generally, Kuntz-type inhibitors with a P1 site of Lys or Arg tend to inhibit trypsin, while chymotrypsin inhibitors possess a P1 site of Leu, Met, Phe, Tyr, Trp, or Asn [1], [9]. Interestingly, AvKTI, which possesses the basic amino acid Lys within the P1 site, shows a dual activity against trypsin and chymotrypsin. A similar result has been demonstrated for a snake venom serine protease inhibitor, which possesses Lys in the P1 site and exhibits dual inhibitory activity against trypsin and chymotrypsin [12]. Additionally, the P1-P1′ residues in AvKTI are Lys and Ala, as has been demonstrated for bovine pancreatic trypsin inhibitor (BPTI), which inhibits trypsin, but it also inhibits chymotrypsin, plasmin, and kallikrein [24], [25]. Collectively, these data indicate that AvKTI is a member of the Kunitz-type serine protease inhibitor family.

Some Kunitz-type serine protease inhibitors exhibit antifibrinolytic activity [7], [15], [17]–[19] and/or antielastolytic activity [8], [15]. We therefore tested whether AvKTI may also target additional serine proteases, such as plasmin and neutrophil elastase. The observed inhibitory activity of AvKTI indicates that AvKTI strongly inhibits plasmin, which degrades fibrin into FDPs [7], [26], [27]. Moreover, it displayed inhibitory activity against neutrophil elastase, which plays a major role in the modulation of thrombosis and fibrinolysis and the regulation of inflammatory signaling [28]. However, AvKTI has no detectable inhibitory effect on factor Xa, thrombin, or tPA, which are enzymes associated with the hemostatic system [7]. Our data clearly show that AvKTI exhibited strong inhibitory activity against plasmin and also inhibited neutrophil elastase, although with a relatively decreased efficiency, indicating that AvKTI targets plasmin and neutrophil elastase. A possible mechanism for the antifibrinolytic and antielastolytic activities of AvKTI may be the formation of a plasmin-AvKTI complex and an elastase-AvKTI complex, as has been demonstrated for serine protease inhibitors, which function by binding to their cognate enzyme, forming a stable complex [7], [29]. The finding that AvKTI inhibits plasmin and neutrophil elastase is similar to previous findings that Kunitz-type protease inhibitors derived from cattle ticks, snakes, black flies, and bumblebees show inhibitory activity against trypsin, plasmin, plasma kallikrein, thrombin, and neutrophil elastase [7], [8], [13], [15], [18]–[20], [30], [31]. Taken together, our results demonstrate that AvKTI acts as a plasmin inhibitor and an elastase inhibitor, suggesting that AvKTI can function as an antifibrinolytic factor and an antielastolytic factor.

Overall, our work provides cloning and functional features of a spider Kunitz-type serine protease inhibitor that exhibits inhibitory activity against trypsin, chymotrypsin, plasmin, and neutrophil elastase. Our results define roles for AvKTI as a plasmin inhibitor and an elastase inhibitor. Given that trypsin or chymotrypsin inhibition and K^+^ channel blocking are known functions of the spider-derived Kunitz-type proteins [3], [16], the inhibitory ability of AvKTI against plasmin and neutrophil elastase appears to be a novel function of spider-derived Kunitz-type serine protease inhibitors. The finding that AvKTI exhibits antifibrinolytic and antielastolytic activities not only highlights the potential roles of spider-derived Kunitz-type proteins, but it will also have significant implications for the future investigations of spider-derived Kunitz-type proteins.

## Materials and Methods

### cDNA Cloning and Sequence Analysis

A clone encoding AvKTI was selected from the expressed sequence tags (ESTs) that were generated from a cDNA library constructed using whole bodies of the spider *A. ventricosus* [32]–[34]. Plasmid DNA was extracted using the Wizard Mini-Preparation kit (Promega, Madison, WI, USA). The cDNA sequence was analyzed using an ABI310 automated DNA sequencer (Perkin-Elmer Applied Biosystems, Foster City, CA, USA). Sequenced cDNA was compared using the DNASIS and BLAST databanks online from NCBI (http://www.ncbi.nlm.nih.gov/BLAST). MacVector (ver. 6.5, Oxford Molecular Ltd., Oxford, UK) was used to align the deduced amino acid sequences of the Kunitz-type inhibitor genes. The signal sequence was predicted by SignalP 4.0 (http://www.cbs.dtu.dk/services/SignalP).

### RNA Extraction and Northern Blot Analysis

Total RNA was isolated from the epidermis, fat body, midgut, and venom gland of *A. ventricosus* using a Total RNA Extraction Kit (Promega). The total harvested RNA (5 mg/lane) was separated using a 1.0% formaldehyde agarose gel, transferred onto a nylon blotting membrane (Schleicher & Schuell, Dassel, Germany), and hybridized at 42°C with the appropriate probe diluted in hybridization buffer containing 5× SSC (0.75 M sodium chloride and 0.75 M sodium citrate), 5× Denhardt’s solution (0.1% each of bovine serum albumin (BSA), Ficoll, and polyvinylpyrrolidone), 0.5% SDS, and 100 mg/ml denatured salmon sperm DNA. *AvKTI* cDNA was labeled with [α-^32^P] dCTP (Amersham Biosciences, Piscataway, NJ, USA) using the Prime-It II Random Primer Labeling kit (Stratagene, La Jolla, CA, USA), and labeled cDNA was used as a probe for hybridization. After hybridization, the membrane filter was washed three times for 30 minutes each in 0.1% SDS and 0.2× SSC at 65°C and then exposed to autoradiography film.

### Expression of Recombinant Protein

A baculovirus expression system [35], using the *Autographa californica* nucleopolyhedrovirus (AcNPV) and the *Spodoptera frugiperda* (Sf9) insect cell line, was employed to construct a recombinant virus expressing AvKTI. The *AvKTI* cDNA, which encoded 57 amino acids as a mature peptide, was PCR-amplified from *pBluescript-AvKTI* using the forward primer 5′-AAAGATAGATGCCTTCTTCCG-3′ and the reverse primer 5′-TTAATGATGATGATGATGATGGTGATCCGTGCAGGCTTTTTCGCA-3′. The reverse primer for the amplification of *AvKTI* was engineered to include His-tag sequences. PCR cycling conditions were as follows: 94°C for 3 min, 30 cycles of amplification (94°C for 30 sec, 55°C for 30 sec, and 72°C for 1 min), and 72°C for 5 min. PCR products were sequenced using the BigDye Terminator Cycle Sequencing Kit and an automated DNA sequencer (Perkin-Elmer Applied Biosystems). The *AvKTI* fragment was inserted into the *pBacPAK8* vector (Clontech, Palo Alto, CA, USA) to generate an expression vector under the control of the AcNPV polyhedrin promoter. The honeybee melittin signal peptide [36] was used as a signal sequence for the secretion of AvKTI. For expression experiments, 500 ng of the construct (*pBacPAK8-AvKTI*) and 100 ng of the AcNPV viral DNA [35] were co-transfected into 1.0–1.5×10^6^ Sf9 cells for 5 h using the Lipofectin transfection reagent (Gibco BRL, Gaithersburg, MD, USA). Transfected cells were cultured in TC100 medium (Gibco BRL) supplemented with 10% fetal bovine serum (FBS, Gibco BRL) at 27°C for 5 days. Recombinant baculoviruses were propagated in Sf9 cells cultured in TC100 medium at 27°C. The recombinant proteins were purified using the MagneHis™ Protein Purification System (Promega). The protein concentrations were determined using a Bio-Rad Protein Assay Kit (Bio-Rad, Hercules, CA, USA). Glycoprotein staining was performed using a Gel Code Glycoprotein Staining Kit (Pierce, Rockford, IL, USA).

### Western Blot Analysis

Western blot analysis was performed using an enhanced chemiluminescence (ECL) Western blotting system (Amersham Biosciences). Protein samples were mixed with sample buffer, boiled for 5 min, and separated using 14% SDS-polyacrylamide gel electrophoresis (SDS-PAGE). Following electrophoresis, proteins were transferred onto a nitrocellulose membrane (Schleicher & Schuell), and then the membrane was blocked in 1% BSA. Then, the membrane was incubated with anti-His antibody at room temperature for 1 h and washed in Tris-buffered saline with Tween-20 (TBST, 10 mM Tris-HCl, pH 8.0, 100 mM NaCl, and 0.05% (w/v) Tween-20). The membrane was then incubated with horseradish peroxidase–conjugated anti-mouse IgG diluted 1∶5,000 (v/v). After repeated washes with TBST, the membrane was incubated with ECL detection reagents (Amersham Biosciences) and exposed to autoradiography film.

### Serine Protease Inhibition Assay

Bovine trypsin (100 nM) (Sigma, St. Louis, MO, USA) or 100 nM of bovine α-chymotrypsin (Sigma) was incubated in 100 mM Tris-HCl (pH 8.0) containing 20 mM CaCl_2_ and 0.05% Triton X-100 with increasing amounts of AvKTI at 37°C for 30 min. The residual enzyme activity was determined at 405 nm or 410 nm using the following substrates: 0.5 mM BApNA (Sigma) for trypsin and 0.5 mM Suc-AAPF-pNA (Sigma) for α-chymotrypsin. Additionally, 100 nM of human plasmin (Sigma), human neutrophil elastase (Sigma), human thrombin (Sigma), human tissue plasminogen activator (tPA; Sigma), or bovine factor Xa (Novagen, Darmstadt, Germany) was incubated with increasing amounts of AvKTI at 37°C for 30 min in 50 mM Tris-HCl buffer (pH 7.4), and the residual enzyme activity was determined at 405 nm using 0.5 mM of the substrates S-2251 (Chromogenix, Mölndal, Sweden) for plasmin, S4760 (Sigma) for neutrophil elastase, S-2238 (Chromogenix) for thrombin, S-2288 (Chromogenix) for tPA, and S-2222 (Chromogenix) for factor Xa [7], [26], [27]. The initial reaction rate was determined by calculating the slope of the linear portion of the kinetic curve. The inhibitory effect was expressed as the percent reduction in the initial hydrolysis rate; the reaction rate in the absence of inhibitor was defined as 100%. The inhibitor concentration that decreased the rate of hydrolysis by 50% (IC_50_) was determined. The values of the inhibition constants (K_i_) were calculated using the equation K_i_ = IC_50_/(1+S/K_m_) [37].

### Fibrinolytic Cleavage Assay

Human fibrinogen (200 µg, Sigma) that had been clotted with 1 unit of thrombin in 50 mM Tris-HCl buffer (pH 7.4) containing 5 mM CaCl_2_ was incubated with plasmin (1000 ng) or both plasmin and AvKTI (50 ng) at 37°C. The fibrinolytic cleavage was analyzed using 14% SDS-PAGE [7].

### Fibrin Plate Assay

The fibrin plate assay was performed with 10 ml of human fibrinogen (0.6%) clotted with three units of thrombin. Plasmin or a mixture of plasmin and AvKTI was dropped onto the fibrin plates, and the plates were incubated at 37°C for various time periods. The fibrinolytic activity was determined by measuring the formation of a clear area on the plates [7], [26], [27].

### Electrophoretic Mobility Shift Assay

The electrophoretic mobility shift assay was performed as previously described [7]. Plasmin (1 µg) or neutrophil elastase (1 µg) in 50 mM Tris-HCl buffer (pH 7.4) containing 5 mM CaCl_2_ was mixed with 0.5 µg of AvKTI and incubated at 37°C for 1 h. Samples were resolved on a 10% polyacrylamide gel at 4°C. Following electrophoresis, the proteins were blotted onto a sheet of nitrocellulose transfer membrane (Schleicher & Schuell). The membrane was blocked by incubation in a 1% BSA solution and then incubated with diluted (1∶1,000 v/v) antiserum against plasmin, neutrophil elastase, or the His-tag at room temperature for 1 h. After washing in TBST, the membrane was incubated with horseradish peroxidase-conjugated anti-mouse IgG diluted 1∶5,000 (v/v). After repeated washing, the membrane was incubated with ECL detection reagents (Amersham Biosciences) and exposed to autoradiography film.
